# Supplementary material for: Mitogen-Activated Protein Kinase Is Involved in Salt Stress Response in Tomato (Solanum lycopersicum) Seedlings
Source: Int J Mol Sci. 2022 Jul 11;23(14):7645. doi: 10.3390/ijms23147645 (PMC9319631; doi:10.3390/ijms23147645)
Supplement: Supplementary file 1 [file ijms-23-07645-s001.zip › Table S1 Statistics and quality evaluation of RNA-seq data.pdf]

Table S1 Quality statistics of transcriptome.

Sample	Raw reads	Clean reads	Clean bases	Error rate	Q20	Q30	GCpct
Control 1	51166330	50308986	7.55G	0.03	97.73	93.4	42.97
Control 2	51423804	50581532	7.59G	0.03	97.79	93.58	42.99
Control 3	52427908	51445114	7.72G	0.03	97.27	92.4	43.13
NaCl 1	55034310	54136256	8.12G	0.03	97.77	93.51	42.77
NaCl 2	56240960	55303674	8.3G	0.03	97.69	93.39	42.85
NaCl 3	45997542	44587798	6.69G	0.03	97.94	93.89	42.65
